# Genetically Engineered Pigs to Study Cancer

**DOI:** 10.3390/ijms21020488

**Published:** 2020-01-13

**Authors:** Daniela Kalla, Alexander Kind, Angelika Schnieke

**Affiliations:** Chair of Livestock Biotechnology, School of Life Sciences, Technische Universität München, 85354 Freising, Germany; kalla@wzw.tum.de (D.K.); kind@wzw.tum.de (A.K.)

**Keywords:** preclinical cancer models, pig, swine, transgenic, pancreatic cancer, colorectal cancer, breast cancer, osteosarcoma, genome editing

## Abstract

Recent decades have seen groundbreaking advances in cancer research. Genetically engineered animal models, mainly in mice, have contributed to a better understanding of the underlying mechanisms involved in cancer. However, mice are not ideal for translating basic research into studies closer to the clinic. There is a need for complementary information provided by non-rodent species. Pigs are well suited for translational biomedical research as they share many similarities with humans such as body and organ size, aspects of anatomy, physiology and pathophysiology and can provide valuable means of developing and testing novel diagnostic and therapeutic procedures. Porcine oncology is a new field, but it is clear that replication of key oncogenic mutation in pigs can usefully mimic several human cancers. This review briefly outlines the technology used to generate genetically modified pigs, provides an overview of existing cancer models, their applications and how the field may develop in the near future.

## 1. Introduction

Human lifespan is continually increasing, as are expectations of health and well-being [1]. There is accordingly greater concern about age-related diseases such as cancer, cardiovascular diseases and diabetes [2]. The overall incidence of cancer is increasing [3] and certain cancers, e.g., pancreatic, urgently require improved diagnosis and treatment. However, the number of approvals for new cancer drugs is lower than for other diseases [4].

Animals have long been studied to gain insight into human diseases and remain an essential part of cancer research. Valuable data can be derived from non-mammalian species such as zebrafish, *Danio rerio*. While principally a model of development, zebrafish have been used to model cancers e.g., liver and pancreatic cancer [5,6,7]. Zebrafish have the interesting advantage that embryos and larvae are naturally transparent and transparent adults can be generated [8,9]. This facilitates the study and tracking of tumor angiogenesis [10], metastasis [11,12], and the evaluation of anti-angiogenic agents [13,14] in vivo. However, zebrafish are very different to humans in size, lifespan and especially environmental factors.

Mice are by far the most frequently used laboratory mammal, mainly because of the ease with which they can be housed, bred, and genetically modified [15]. They have provided a wealth of knowledge regarding the molecular and genetic bases of many human cancers and facilitated many proof-of-principle studies. Their short gestation time and relatively inexpensive upkeep are clear advantages over larger animal species, but their usefulness for preclinical research also has limitations.

They differ considerably from humans in size, lifespan and aspects of organ anatomy, e.g., pancreas and spleen [16,17] and in the ease with which murine cells undergo oncogenic transformation compared with human cells [18,19].

Furthermore, replication of human oncogenic mutations in mice can fail to recapitulate the human pathology [20], and murine tumors differ in important clinical features such as drug response, possibly due to differences in immunology and drug metabolism [21,22].

The murine innate and adaptive immune systems differ significantly from humans, leading to different responses in inflammatory disease that can affect cancer development [23,24].

Consequently, many of the drugs evaluated in mice fail in clinical trials [25]. There is thus a need for non-rodent species to provide complementary data and improve the predictive value of preclinical studies.

## 2. Large Mammals as Biomedical Models

**Figure 1:**
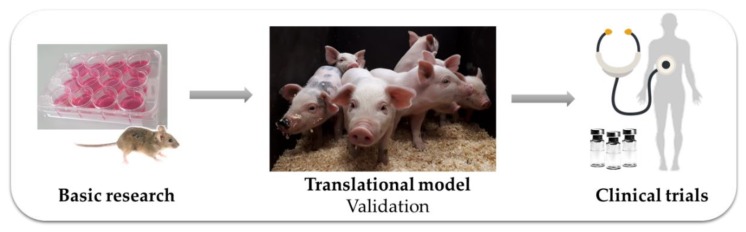
The pig as a biomedical model. Pigs can help translate basic research findings into new medical drugs and procedures; ‘bridging the gap between bench and bedside’.

Larger animals, such as dogs, cats, non-human primates, and pigs each share some similarities with humans and have been considered as research species. Dogs and cats spontaneously develop tumors [26,27], and their veterinary treatment has provided valuable information for cancer research, but the use of companion animals in systematic experimental research is not well accepted by the general public. Similarly, strict regulatory requirements and ethical concerns restrict the use of non-human primates. However, pigs have been domesticated for centuries as a food source and their humane, ethical use as experimental animals under regulated conditions raises fewer concerns [28]. Pigs share many similarities with humans in body size, organ size and architecture, physiology and pathophysiology, and are thus a valuable species for biomedical research [29,30]. Pigs have long been used to study the effect of nutrition, to assess new surgery procedures or improve organ transplantation and for the development of imaging modalities where human scale equipment can be used. Moreover, their relatively long lifespan of 12–15 years [31] allows longitudinal studies to be carried out to assess or validate novel biomarkers, treatment or imaging options, follow disease progression and regression in a single animal [32]. For drug trials, pigs also show similar pharmacokinetic responses to humans [33,34]. Thus, pigs are recognized as a useful animal model for translational medicine (Figure 1).

The use of pigs in biomedicine does have disadvantages relative to smaller species, mainly because of the space required for housing and the time needed for breeding. Veterinary care and handling are also rather different to small laboratory mammals such as rats and mice, but pigs are a very common agricultural species so veterinary expertise is widely available and husbandry procedures well established. Perhaps the greatest difficulty has been in engineering precise genetic modifications into pigs but, as outlined in the next section, this is rapidly changing.

## 3. Generation of Genetically Modified Pigs

Genetic modification of large mammals began when Palmiter and Brinster produced transgenic rabbits, sheep and pigs by microinjection of DNA into the pronuclei of fertilized oocytes [35]. This procedure however resulted in a low proportion of transgenic offspring, usually 1–5% [36]. The high lipid content of porcine oocytes also makes it difficult to visualize the pronuclei. The generation of excess non-transgenic animals, often greater than 95%, was ethically and practically undesirable. Furthermore, DNA microinjection as originally conceived, enables only the addition of transgenes at random locations in the host genome. This prompted a search for more efficient and versatile methods.

The tremendous success of gene targeting in ES cells [37,38] revolutionized reverse genetics in mice and since led to a profusion of modified mouse lines. The potential usefulness of an equivalent method of transferring predetermined genetic modifications from cultured cells to lines of pigs has long been evident. However, despite numerous attempts over decades, the isolation and culture of definitive porcine ES cells, i.e., capable of populating the germline, had been unsuccessful [39,40]. This may now change, porcine expanded pluripotent stem cells (EPSCs) have recently been reported capable of forming all three germ layers in chimeric animals, but germ line contribution is still elusive [41].

The development of nuclear transfer from somatic cells grown in culture [42] provided the first alternative means of generating genetically modified livestock. Using in vitro transfected primary cells as nuclear donors increased the proportion of transgenic animals to virtually 100% [43] and, importantly, enabled gene targeting in mammals other than mouse [44]. While first established in sheep, nuclear transfer was soon extended to pigs for random transgenesis [45] and targeted gene knockout [46]. However, the procedure is technically challenging and very few lines of viable gene-targeted pigs were generated during the subsequent decade.

The use of tailor-made highly-specific endonucleases has now raised the efficiency of gene targeting. The well-known RNA-guided CRISPR-Cas9 endonuclease system is currently the method of choice, superseding earlier tools such as zinc-finger nucleases and TALENs [47,48]. Gene editing was quickly used in pigs to effect genetic alterations, including gene inactivation via small insertion/deletions (indels) as a consequence of non-homologous end-joining [49,50,51], or sequence replacement via homology-directed repair [52].

Inspired by work originated in mice [53] transgenic pigs have recently been generated that express a Cas9 transgene placed at the *ROSA26* locus in a Cre-dependent manner [54], or ubiquitously throughout the body (own unpublished work). This enables gene editing of somatic cells in vivo by local delivery of single or multiple guide RNAs with or without Cre-recombinase. In vivo genome editing is still in its early stages, but offers a very powerful tool for controlled and efficient genome modification in defined organs and cell types at any age. For example in modelling cancer, tumor-supportive and -suppressive genes can be modified in successive rounds of gene editing to mimic the accumulation of mutations that accompany the progression of tumor entities [55]. Somatic modification also allows otherwise lethal or deleterious mutations to be studied in particular tissues or organs free of the complicating effects in the rest of the organism.

In practical terms, Cas9-expressing animals can reduce the time involved in generating and breeding new lines carrying mutations of interest, a significant advantage for larger species.

Site-specific recombination systems e.g., Cre/loxP or FLP/FRT have long been established in the mouse as a means of removing transcriptional stop cassettes to activate Cas9 or latent oncogenic alleles at chosen locations and even at a chosen time [56]. At the time of writing no pig line has yet been established that expresses Cre-recombinase in a tissue-specific manner.

This is clearly a deficiency, but efforts are underway to establish both Cre transgenic animals and also devise efficient means of delivering Cre, as DNA or protein, directly to the organ of choice.

Transgenic pigs that express a Cre-responsive dual fluorescent reporter provide an important tool to establish and assess the success of these methods [57]. Regarding the delivery of guide RNAs, numerous methods from viral vectors to nanoparticles are currently being developed in the mouse and will undoubtedly soon be transferred to the large animals.

These technical advances are central to the success of modelling human diseases in pigs. Some examples include skin wound healing [58,59]; modelling neurodegenerative diseases such as Alzheimer’s [60,61] and Huntington’s disease [62]; cardiovascular diseases [63,64], diabetes [65,66]; and monogenic diseases including Duchenne muscular dystrophy and cystic fibrosis [67,68]. These will increasingly inform how pigs can best contribute to preclinical studies, much as happened with mice over the past decades.

## 4. Porcine Cancer Models

The value of pig cancer models obviously depends on how faithfully they represent human disease. Porcine cancer biology is still a new field, but indications are that pigs can correctly mimic human cancers. Spontaneous cancers occur only rarely in wild type pigs and, as in humans, arise mostly with age [20]. Similar to humans, oncogenic transformation of porcine cells is a rare event that requires multiple genetic alterations [69]. A fundamental question has been whether replication of human oncogenic mutation(s) in a pig has an equivalent effect on cell transformation and tumorigenesis. So far, this does appear to be the case. Adam and colleagues introduced sets of overexpressed oncogenic transgenes into porcine primary fibroblast cells, which were tumorigenic when returned to the donor animals by autologous transplantation [70]. Our group has systematically investigated the stages of sarcomagenesis in vitro and found that porcine mesenchymal stem cells (MSCs) resemble human MSCs in that they require perturbation of p53, KRAS and MYC signaling pathways with spontaneous Rb pathway inactivation and telomerase-independent immortalization steps to convert to a fully transformed phenotype [71]. This contrasts with murine MSCs that can be transformed by loss of p53 function alone [72].

These findings suggest basic similarity between porcine and human oncogenesis, but in vitro culture, randomly integrated overexpressed transgenes and engraftment of transformed cells can all be criticized as artificial non-physiological methods. In our view, the generation of autochthonous tumor entities by replication of oncogenic lesions in endogenous porcine genes is the ‘gold standard’ and cancer models generated in this way are likely to be the most representative of human disease. In the examples given below both types of models will be presented and compared.

Table 1 provides an overview of genetically modified pig models for human cancers.

### 4.1. Porcine Models for Breast Cancer

Breast cancer is a common form of cancer and the leading cause of cancer-related death among women worldwide [85]. Despite tremendous advances in the past, incidence rates have been steadily increasing in the last decade [86]. Approximately 5–7% of all cases are diagnosed in women younger than 40 years old [87,88], whose disease progression is often more aggressive than in older women [89]. Breast cancer in young people is more often associated with germline mutations in the *BRCA1/2* genes which constitute an increased familial risk for breast and ovarian cancer [90,91]. Indeed, the median ages at diagnosis for carriers of *BRCA1* or *BRCA2* mutations is 40 and 43 years [92]. BRCA1 and 2 are tumor suppressors that play an essential role in homologous repair of DNA breaks [93]. Thus, mutations in *BRCA1/2* lead to genomic instability and predisposition to cancer [94].

Breast cancer was the subject of the first attempt to model a human cancer in genetically modified pigs. Nagashima and colleagues reported pigs carrying a v-Ha-ras oncogene with expression directed to mammary epithelium by a murine mammary tumor virus promoter, but observed no phenotype [73]. Transgene expression was detected in tissues such as lung and spleen, but was absent in mammary gland. This might have been due to a position effect of the randomly placed transgene cassette and/or silencing of the viral promoter by methylation. A later attempt aimed to inactivate the endogenous porcine *BRCA1* locus, but heterozygous *BRCA1* knockout piglets were inviable, whether due to the mutation or defects from nuclear transfer is not known [74]. Inactivation of *BRCA1* in a porcine mammary cell line does result in a transformed phenotype resembling human breast cancer, suggesting that the pig is a suitable species to model breast cancer [95], but a representative pig model has yet to be produced.

Breast cancer remains such an important and common disease that further efforts are undoubtedly required. Indeed, the mammary gland is relatively accessible in living animals and would be a good candidate for in vivo gene editing using Cas9-expressing pigs, as described above. This would enable local inactivation or modification of key initiating genes such as BRCA1 and 2, and genes involved in disease progression e.g., TP53, and PIK3CA [96] without affecting overall animal development or viability.

### 4.2. Porcine Models for Colorectal Cancer

Colorectal cancer (CRC) is the third most common human cancer worldwide, and was the second leading cause of cancer-related deaths in 2018 [85]. While CRC incidence has decreased in patients older than 50 years, mostly due to routine screening, there has been an alarming increase in people under 50, and by 2030, colorectal cancer is expected to increase by more than 90 percent in people aged 20–34 years [97,98,99,100].

Colorectal cancer arises from the epithelial lining of the colon and rectum, with functional disruption of the tumor suppressor adenomatous polyposis coli (*APC*) the main event that initiates formation of adenomatous polyps [101,102]. As part of the β-catenin destruction complex, APC acts as a negative regulator of the Wnt pathway [103]. Loss or dysfunction leads to aberrant Wnt signaling resulting in increased proliferation and tumor formation [104]. Progression to cancer involves additional mutations and genomic instability [105].

In sporadic CRC, somatic *APC* mutations mainly occur in the mutation cluster region between codons 1281 and 1556 [106,107]. Germline mutations are found throughout the 5′ part of the gene, with two common hot spots at codons 1061 and 1309 [108], and are responsible for familial adenomatous polyposis (FAP), a hereditary predisposition for CRC that leads to the formation of large numbers of adenomatous polyps in the colon and rectum and adenomas at a young age [109]. If not removed in time, these premalignant lesions can turn into invasive adenocarcinoma.

Many mouse models have been generated to replicate human FAP. However, these have revealed that mutation of *Apc* alone is not sufficient to mimic the human phenotype in mice. The most commonly used *Apc^Min/+^* mouse develops polyps predominantly in the small intestine rather than in the colon [110]. Successful FAP modelling in mice requires more complex modifications with tissue-specific *Apc* deletion and additional mutations [111,112,113].

**Figure 2:**
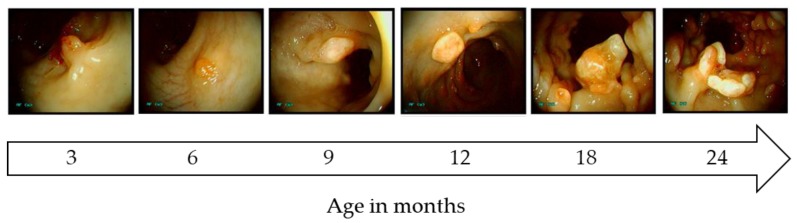
Phenotype of adenomatous polyposis coli (APC)^1311^ pigs. Polyp progression during the first 24 months.

Pigs have been generated that carry a translational stop codon at position 1311 in the endogenous porcine *APC* gene (*APC*^1311^), orthologous to a human *APC*^1309^ mutation responsible for a severe form of FAP [75]. Within their first year *APC*^1311*/+*^ pigs develop polyps in the colon and rectum (Figure 2) that show features typical of the human adenoma-carcinoma sequence, such as aberrant crypt foci and adenomas with low- and high-grade neoplasia and carcinoma in situ [75]. Porcine adenomas exhibit genetic and biochemical hallmarks of human FAP and sporadic CRC, such as loss of the wild type *APC* allele, β-catenin accumulation, high expression of its target gene *c-MYC* and mitogen-activated protein kinase (MAPK) pathway activation [75,114]. These results resemble the findings in human patients, where overexpression of *c-MYC* ensures tumor growth via metabolic reprogramming and survival of colon cancer stem cells [115,116].

An important advantage of initiating precancerous tumors from a single mutation such as *APC*^1311^ is that subsequent spontaneous events leading to cancer can be followed in detail, which is not possible in mice where additional engineered mutations are necessary to ‘force’ disease progression. For example, porcine polyps have been found to show microRNA dysregulation between low- and high-grade dysplasia [117], a natural feature of human cancer progression [118]. *APC^1311/+^* pigs are also being used for preclinical studies, for example to evaluate the use of biodegradable fluorescent nanoparticles to visualize very early adenomas [119].

Others have also attempted to replicate colon cancer in pigs. Pigs with *APC* truncated by a premature stop codon at position 902 have been generated using TALENs, but no phenotype or polyposis has been reported [76].

Pigs with tissue-specific and 4-hydroxytamoxifen (4-OHT)-inducible expression of the oncogenic transgenes *KRAS^G^*^12*D*^, *cMYC,* and *SV40LT* have been reported [77]. However, these pigs developed duodenal carcinoma rather than colon cancer, likely because the oncogenes were overexpressed from a random transgene and activation was regulated by the epithelial villin promoter which would be expected to drive expression throughout the whole intestine.

To date the *APC*^1311*/+*^ pigs are the only model carrying an endogenous mutation that leads to formation of polyposis in the colon and rectum. Due to their size and long lifespan, disease progression and mutation accumulation can be monitored by screening via colonoscopy. Ongoing studies are investigating the effects of diet and involvement of the microbiome in disease progression. One drawback of the porcine model is the slow progression to invasive cancer, as in humans. Artificial acceleration to late stage disease can however be engineered by introducing additional oncogenic mutations by breeding or in vivo genome editing of individual polyps.

### 4.3. Porcine Models for Pancreatic Cancer

Pancreatic cancer is the 11th most common cancer worldwide [85], but is a leading cause of cancer-related deaths due to the overwhelmingly poor prognosis [120]. Alarmingly, incidence is increasing and pancreatic cancer is expected to surpass colorectal cancer and breast cancer to become the second leading cause of cancer-related deaths in Germany and the United States by 2030 [121,122].

Pancreatic cancer mainly arises from the exocrine component, with less than 5% of all tumors developing from endocrine cells [123]. Pancreatic ductal adenocarcinoma (PDAC) accounts for more than 90% of exocrine malignancies. Several precursor lesions for PDAC have been described with proliferating epithelial lesions, pancreatic intraepithelial neoplasia (PanIN), the most prominent [124]. The main driver of PanIN formation is activation of the proto-oncogene *KRAS*, which is mutated in more than 90% of all PDAC cases, mostly a G to D amino acid substitution at codon 12 [125]. Progression of PanINs to PDAC is associated with accumulation of mutations in the tumor suppressor genes *CDKN2A (p16)*, *TP53*, *SMAD4* and *BRCA1/2* [126].

PDAC was previously thought to derive solely from the epithelial lining of the pancreatic duct, but evidence now suggests acinar cells that undergo transdifferentiation to a ductal-like phenotype, acinar-to-ductal metaplasia (ADM). ADM is a reprogramming phenomenon that can be a consequence of stress and inflammation such as pancreatitis [127,128]. Indeed, PDAC has been shown to arise from both acinar and ductal cell types, but it is believed that acinar-derived PDAC develops via PanINs, while ductal-derived PDAC develops in a PanIN-independent manner [129,130].

The high morbidity of PDAC can be ascribed to several characteristics of the disease. Perhaps the most important is that it is aggressive and metastatic even at early stages, but is usually asymptomatic. Diagnoses tend to be made only when the tumor is advanced and unresectable due to complex vascular invasion and metastasis is in progress. Newly diagnosed patients thus have a five-year survival rate of only 9% [131]. While there are some reports of successful treatment of locally advanced PDAC by surgery and chemotherapy [132], such cases are rare. PDAC is also characterized by high intra-tumoral heterogeneity and plasticity that foster the emergence of drug-resistant populations that render most conventional therapies ineffective [133,134,135].

The search for better early diagnosis and effective treatments has motivated the generation of several mouse models to mimic human PDAC. The genetic requirements for PDAC development were defined in a series of key studies by Tuveson and colleagues. They reported that expression of *Kras^G^*^12*D*^ directed by either the *Ptf1a* or *Pdx1* promoters resulted in PanIN lesions, but these rarely developed to invasive carcinoma [136]. In contrast, combination of *Kras^G^*^12*D*^ with deletion of *Cdkn2a* led to aggressive tumors that invaded other organs. This however resulted in death at 11 weeks, preventing the timely formation of distant metastases [137].

Most usefully it was shown that Pdx1-promoter-directed expression of *Trp53^R^*^172*H*^ in combination with *Kras^G^*^12*D*^, orthologues of the most common mutations present in human PDAC, initiated the development of a widely metastatic PDAC in mice that recapitulated the main characteristics of the human disease [138].

The success of the mouse work has prompted efforts to generate similar models in pigs. Schook et al. have generated pigs with random transgenes containing Cre-inducible *KRAS^G^*^12*D*^ and *TP53^R^*^167*H*^ mutations (orthologous to human *TP53^R^*^175*H*^) driven by the CAG-promoter [80]. Explanted cells from these animals transduced with adenovirus encoding Cre (AdCre) in vitro became transformed, while subcutaneous and intramuscular injection of AdCre led to tumor formation in vivo [80]. Interestingly, these pigs revealed marked intra-tumoral T-cell infiltration and an anti-tumor immune response regardless of the site of tumor formation [139]. These findings suggest that pig tumors are subject to strong immune surveillance, making them suitable to test possible immunotherapies. With regard to pancreatic cancer, AdCre administered to pancreatic duct cells in vitro render them immortal and capable of forming tumors when injected in immune-deficient mice [81]. Delivery of AdCre into the main porcine pancreatic duct in vivo gave rise to tumors that displayed features of human PDAC, e.g., a dense tumor stroma and E-cadherin expression. However, the pancreatic tumors also contained areas with neuroendocrine rather than PDAC phenotype [81], suggesting activation of the mutant transgenes in a variety of cell types. This is perhaps a consequence of non-specific viral transduction and the constitutively active CAG-promoter. Furthermore, these pigs showed no clinical symptoms, and tumors were not detectable by computer tomography one year after injection, but were found in the pancreatic duct upon resection [81]. The relevance of this model for human PDAC is thus not clear and perhaps illustrates the drawbacks of adding transgenes rather than modifying endogenous genes. Adding a transgene is necessarily artificial and can be non-physiological in some important respects. Normal gene dosage is disturbed because two endogenous non-mutant alleles are still in place. Transgenes placed at random locations are not subject to the normal regulatory influences and, depending on the constructs, can be expressed at artificially high levels.

Another group has reported a porcine model for pancreatic cancer based on overexpression of a multi-oncogene cassette consisting of *KRAS^G^*^12*D*^, *cMYC* and *SV40LT* [82]. In contrast to the models of Schook and colleagues, oncogene expression was induced during embryogenesis using the murine *Pdx1* promoter. None of the transgenic piglets survived long, most likely due to the cloning procedure. Pancreatic acinar cells of one piglet showed hyperplastic foci with colocalized oncogene expression and increased proliferation at day 45 after birth [82]. As *Pdx1* is expressed throughout the whole pancreas during embryogenesis [140], and later becomes restricted to β-cells [141], oncogene activation would not be limited to acinar cells. The presence of hyperplastic foci in the acinar cell compartment could indicate PDAC development via ADM, but would require further investigation. 

Another strategy used to generate a porcine PDAC model is orthotopic xenotransplantation of transformed cells. Explanted pancreatic ductal epithelial cells transformed by overexpression of *KRAS^G^*^12*D*^ and *TP53^R^*^167*H*^, and knock down of *p16* and *SMAD4* have been transplanted into the pancreas of immune-deficient mice. This resulted in the formation of metastatic tumors [142]. Implantation of ex vivo transformed cells into the pancreas of pigs has not yet been performed.

As this model uses transformed pancreatic ductal cells, the resulting tumors are likely to originate from this cell type [143], removing the uncertainty inherent in the transgenic models above. However, allogeneic implantation into pigs can lead to immune rejection and such tumors obviously originate in a quite different manner to spontaneous pancreatic cancers. Indeed, tumors from engrafted cells often derive from one or a few dominant cell clones and are thus less likely to recapitulate important features such as tumor heterogeneity.

Work towards another model has been based directly on Tuveson’s PDAC mice [138]. Schnieke and colleagues reported the first generation of pigs with latent *KRAS^G^*^12*D*^ and *TP53^R^*^167*H*^ mutations engineered into the endogenous genes [78,79]. As in the equivalent murine alleles [136], expression is blocked by a floxed transcriptional stop cassette and activated by Cre recombination. Pigs carrying the latent *KRAS^G^*^12*D*^ and *TP53^R^*^167*H*^ alleles in heterozygous form are viable and can be bred normally although, as described later, uninduced *TP53^R^*^167*H*^ animals develop osteosarcoma in later life. This is a multi-component system that requires some means of specifically expressing Cre-recombinase in pancreas to activate the latent *KRAS^G^*^12*D*^ and *TP53^R^*^167*H*^ alleles. This is not yet in place, but methods are being employed to deliver Cre locally into the porcine pancreas, and to use pancreatic promoters including PDX1 and PTF1A to direct expression. This work is aided by the use of a dual-fluorescent reporter pig that enables cells that have undergone Cre-recombination to be visualized by a switch in fluorescence [57]. While still incomplete this model has the advantage over the transgenic models that it should more closely mimic the events that cause spontaneous human PDAC.

### 4.4. Porcine Models for Osteosarcoma

Osteosarcoma (OS) is the major form of primary bone cancer, and is commonly located in the metaphyseal growth plates of the long bones of the extremities [144]. It predominantly affects young people and is highly malignant, requiring aggressive surgical resection and cytotoxic chemotherapy [145]. The 5-year survival rate has remained unchanged for decades, at ~60% for patients with primary OS and ~20% for patients with metastatic disease [146].

Most cases of human OS are sporadic, with identified risk factors that include rapid bone growth, exposure to radiation, and genetic diseases [144]. Increased incidence of OS is associated with Li-Fraumeni syndrome caused by germ line mutation of *TP53* [147] and hereditary retinoblastoma caused by germ line mutation of *RB1* [148,149].

Human OS displays high rates of chromosomal alterations and structural changes [150,151], and an overwhelming prevalence of mutations affecting p53 function [152,153]. While alterations have also been found in other genes including *RB1*, *ATRX*, and *DLG2* [153], *CDKN2A/B* [154], *PTEN* [155], *IGF1R* [156], and several genes in the PI3K/mTOR pathway [155], defects affecting p53 predominate.

Three models of OS have been developed. Genetically engineered animal (mouse and pig) models, patient-derived primary tumor cells, and large breed dogs with spontaneous disease [28,157]. The patient-derived human cells and dog OS models are described in a recent review [157]. Of the genetically engineered models, OS development in mice was achieved by inactivating *Trp53* and *Rb1* [158]. Some *Trp53* knockout mice developed OS, but the majority (75%) of mice homozygous for this mutation developed lymphomas [159,160]. Improved mouse OS models have been developed based on conditional *Trp53* inactivation [161], and conditional activation of *Trp53* hot spot mutations in the osteogenic lineage [162]. These show highly penetrant OS formation, mainly in the axial skeleton, a location rarely observed in human OS [163].

Two pig lines that develop OS have been described. Sieren et al. generated Yucatan minipigs that carry a R167H mutation in the endogenous *TP53* gene that is ubiquitously expressed from the major P1 promoter [83]. They reported that heterozygous *TP53^R^*^167*H*^ mutant pigs showed no tumor development even at 30 months of age, while those homozygotes that reached sexual maturity developed a variety of neoplastic lesions, including osteogenic tumors, lymphomas and renal tumors, broadly recapitulating the tumor spectrum observed in mice with the orthologous mutation.

As described in the section on pancreatic cancer, Schnieke and colleagues have generated pigs that carry a latent *TP53^R^*^167*H*^ mutation in exon 5 that can be activated by Cre-recombination [78]. Observation of pigs over several years has revealed that pigs heterozygous and homozygous for the uninduced allele all develop OS [71]. Compared to the CRC model, the bone tumors develop rapidly in the homozygous animals, as early as six months old. Wilm’s tumors and lymphomas also occur occasionally. Porcine OS displays several similarities with the human disease. As in humans, it primarily affects the long bones, tumor cells show a highly abnormal karyotype and nuclei with atypical mitotic figures, and increased resistance to radiation [71]. The origin of human osteosarcoma is not well understood and these pigs provide a valuable resource to study the underlying mechanisms, an important step towards identifying possible drug targets. They also offer a resource to surgeons and clinicians seeking to improve surgical treatment and maintenance of this devasting condition.

## 5. Porcine Tumor Xenograft Models

Modelling human cancers by xenotransplantation of human cancer cells is well established in mice. Patient-derived xenograft (PDX) mouse models generated by subcutaneous or orthotopic grafting of human tumor samples into severe combined immunodeficient (SCID) mice [164,165] are currently preferred over xenografting of cell lines because those may have lost the original tumor heterogeneity during long periods in culture [166]. The PDX approach is thus a better predictor of human tumor behavior, and murine PDX models have been generated for colon, pancreatic and breast cancers [167,168,169].

Work is proceeding towards producing immunodeficient pigs that would enable a similar xenograft system. Nakayama and colleagues have generated immunodeficient pigs by removing the thymus and spleen in combination with drug immunosuppression [170]. Although effective, this approach is highly invasive and suitable for producing only small numbers of immunodeficient pigs. A better alternative is germline modification of genes required for B- and T-cell development, such as the X-linked interleukin-2 receptor gamma chain gene (*IL2RG*) or V(D)J recombination-activating genes (*RAGs*). Some groups have reported SCID pigs by disruption of *IL2RG* [171,172,173]. These animals lacked a thymus and showed loss or reduction of T- and NK-cells, but survival was poor, mostly due to infections such as pneumonia. Nevertheless, the pigs generated by Onishi and colleagues were subjected to allogeneic bone marrow transplantation and three survived for longer than 300 days [171]. Pigs with disruption of *RAG1* and/or *RAG2* also showed a SCID phenotype lacking T- and B-cells [174,175,176,177]. Kim and colleagues showed that injecting human iPS cells resulted in teratoma formation in *RAG2*-deficient pigs, demonstrating their value [175]. *IL2RG* and *RAG2* double knockout pigs have also recently been reported [177].

The availability of SCID pigs will not only enable human cancer xenograft experiments, but will also allow transplantation of human immune cells to produce “humanized” pigs that can be used for drug and therapy testing [20]. However, immunodeficient pigs are necessarily more susceptible to infection and require pathogen-free housing.

Another means of avoiding immune rejection of tumor cells is based on xenografting into recipient animals in utero. Human cells have been injected into porcine fetuses before CD3+ lymphocytes populate the thymus [178,179]. Human hepatic cells injected into porcine fetal liver have been shown to engraft successfully [178], a process that can be used to tolerize a pig in preparation for further human cell transplantation after birth [20].

## 6. Future Perspectives and Challenges

**Figure 3:**
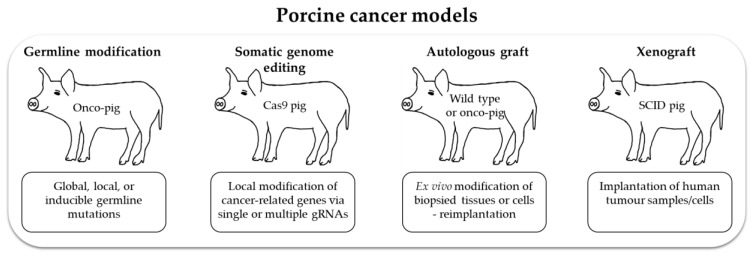
Porcine cancer models. These can be established by different methods: e.g., introduction of oncogenic germline mutations or via genome editing in organs of a Cas9 pig. Biopsies taken early in life can be oncogenically transformed and reimplanted into the same pig. If isolated from a pig with latent oncogenic mutations, these can be activated in vitro prior to implantation. Human tumor samples can also be implanted in immune deficient animals.

Pigs are a relatively new species in which to study cancer. Their value for biomedical research will benefit from continuing increases in physiological, biochemical, immunological and genetic information, and the generation of new models is set to be simplified by improved techniques to modify the germline and somatic cells (Figure 3).

Future research will clearly involve the production of new pig lines, but there is also much to be gained by integrating the study of existing models with advanced culture systems. Tissues can be explanted and cultured as organoids, or grown in air–liquid interface culture systems. Culture systems that mimic the natural three-dimensional tissue organization and location in vivo will enable a wide range of manipulations and investigations to be carried out over relatively short timescales. These could include engineering panels of genetic modifications, coculture with microbiota, and exposure to drugs. Then, because pigs are long lived, explanted cells can be returned to the donor animal as autologous grafts and the effects studied in whole animals (Figure 3). For example, biopsy samples of adenomatous polyps from an *APC*^1311^ pig can be manipulated and characterized in vitro and then reimplanted to study cancer progression. Alternatively, in vivo genome editing of individual polyps can be performed by introducing different mutations into different polyps. Thus, both methods allow multiple experiments in a single animal to address complex questions. In addition, the Cas9-expressing pigs can be cross bred with *APC*, *KRAS* or *TP53* mutant pigs to introduce additional mutations in later life.

The use of animals in biomedical research can be controversial, but there are currently no other means of studying disease in the context of the whole organism, such as interactions with the immune system. Researchers have a duty to ensure that animals are used as efficiently as possible. This means minimizing the numbers necessary to generate genetic modifications, and ensuring that animal models produce high quality predictive information relevant to human patients. Recent years have seen significant progress and we are confident that this will continue.

## Figures and Tables

**Table 1:** Providing an overview of genetically modified pig models for human cancers.

Human Cancer	Genetic Modification	Generated By	Comments	Reference
**Breast Cancer**	MMTV/v-Ha-ras (transgene)	Microinjection	No phenotype	[73]
	Heterozygous *BRCA1* knockout (endogenous gene)	Gene targeting via AAV + SCNT	No survival of born piglets	[74]
**Colorectal Cancer**	Heterozygous *APC*^1311^ truncation mutation (endogenous gene)	Gene targeting + SCNT	Colonic polyposis	[75]
	Heterozygous *APC*^902^ truncation mutation (endogenous gene)	TALENs + Chromatin transfer	No phenotype	[76]
	Flp-inducible KRAS^G12D^ + cMYC+SV40LT (transgenes)	Random integration + SCNT	Villin-driven; Duodenal carcinoma	[77]
**Pancreatic Cancer**	Cre-inducible *TP53^R^*^167*H*^ + *KRAS^G^*^12*D*^ mutation (endogenous genes)	Gene targeting + SCNT	Pancreas-specific activation intended	[78,79]
	Cre-inducible TP53^R167H^ + KRAS*^G^*^12D^ mutation (transgenes)	Random integration + SCNT	AdCre delivery into duct led to tumor formation	[80,81]
	Flp-inducible KRAS^G12D^ + cMYC+SV40LT (transgenes)	Random integration + SCNT	Pdx1-driven; Hyperplastic foci of acinar cells	[82]
**Osteosarcoma**	Hetero- and homozygous knockout of *TP53* major transcript (endogenous gene)	Gene targeting + SCNT	OS primarily affecting long bones	[71]
	Homozygous *TP53^R^*^167*H*^ mutation (endogenous gene)	Gene targeting via AAV + SCNT	Various lesions, e.g., osteogenic tumors, lymphomas and renal tumors	[83]
**Other Cancers**	Human Gli2 transcriptional activator K5-hGli2ΔN (transgene)	Random integration + SCNT	Basal cell carcinoma-like lesions; infection	[84]
	Cre-inducible *TP53^R^*^167*H*^ + *KRAS^G^*^12*D*^ mutation (endogenous genes)	Gene targeting + SCNT	Suitable for diverse cancers, e.g., lung cancer	[78,79]

AAV: adeno-associated virus; SCNT: somatic cell nuclear transfer; AdCre: adenovirus encoding Cre.
